# Cellular-resolution 3D virtual histology of human coronary arteries using x-ray phase tomography

**DOI:** 10.1038/s41598-018-29344-3

**Published:** 2018-07-20

**Authors:** William Vågberg, Jonas Persson, Laszlo Szekely, Hans M. Hertz

**Affiliations:** 1 Department of Applied Physics, KTH Royal Institute of Technology/Albanova, Stockholm, Sweden; 2 Karolinska Institutet, Division of Cardiovascular Medicine, Department of Clinical Sciences, Danderyd University Hospital, Stockholm, Sweden; 3 Laboratory of Clinical Pathology and Cytology, Karolinska University Hospital, Stockholm, Sweden; 4 Department of Pathology, Huddinge, Karolinska Institutet, Stockholm, Sweden

## Abstract

High-spatial-resolution histology of coronary artery autopsy samples play an important role for understanding heart disease such as myocardial infarction. Unfortunately, classical histology is often destructive, has thick slicing, requires extensive sample preparation, and is time-consuming. X-ray micro-CT provides fast nondestructive 3D imaging but absorption contrast is often insufficient, especially for observing soft-tissue features with high resolution. Here we show that propagation-based x-ray phase-contrast tomography has the resolution and contrast to image clinically relevant soft-tissue features in intact coronary artery autopsy samples with cellular resolution. We observe microscopic lipid-rich plaques, individual adipose cells, ensembles of few foam cells, and the thin fibrous cap. The method relies on a small-spot laboratory x-ray microfocus source, and provides high-spatial resolution in all three dimensions, fast data acquisition, minimum sample distortion and requires no sample preparation.

## Introduction

Ischemic heart disease is the leading cause of death in the world^1^. The disease develops slowly (decades) through chronical inflammation and accumulation of lipids in the vascular wall, forming atherosclerotic plaques^2^. Myocardial infarction is a severe fast (minutes) event, resulting atherosclerotic plaque rupture, erosion or calcified nodule, causing thrombosis in the coronary artery^3,4^. Plaques prone to rupture are typically characterized by inflammation, and a necrotic lipid core covered by a thin fibrous cap^5^. The detailed mechanisms behind the formation of atherosclerotic plaques and plaque rupture are still subject to intense research^6^.

The present knowledge about myocardial infarction and plaque rupture is largely based on autopsy studies^4,5,7^. In such studies classical histology is the golden standard for tissue characterization and disease assessment. It provides the necessary cellular resolution, down to the sub-micron level, but is unfortunately destructive and does not preserve sample shape and the most important structural components such as lipids and calcium deposits^8^. In addition, histology is time consuming due to the many process steps (e.g., excision, fixation, dehydration, infiltration, embedding, rehydration, sectioning, and staining) before the visible microscopy, making it less practical for surveying large sets of samples. Furthermore, it only provides high resolution in two dimensions since the resolution in the third dimension is limited by the sectioning, which can be several millimeters thick. Consequently, many micro-anatomical features that could affect the pathological assessment may pass unobserved. Finer sectioning increases the risk of sample damage as well as the total time spent on each sample. Thus, a direct and rapid imaging method for histology-like assessment of coronary artery samples with high spatial resolution in all three dimensions would be valuable. Preferably, the method should be a laboratory tool that allows in-house and direct assessment of, e.g., structure, composition, and vulnerability of plaques.

X-ray micro computed tomography (“micro-CT”) can provide micron-resolution non-destructive 3D imaging. It has been applied to human autopsy samples of coronary arteries^9–11^. However, due to the absorption contrast of micro-CT, high spatial resolution imaging is typically restricted to calcium rich plaques while soft-tissue features can only be detected with low resolution. For example, lipid-rich lesions provide low contrast to the surrounding tissue and are therefore difficult to observe unless they are very large. Micro-CT on carotid artery samples show similar properties^12,13^. However, x-ray phase-contrast methods have demonstrated much better soft tissue contrast^14^, and have enabled improved imaging and soft-tissue differentiation both in human coronary arteries^10,15–17^ and in human carotid arteries^16,18–20^. These studies rely on grating-based phase-contrast images (GBI, cf. next section), both at synchrotron sources and at laboratory sources. The observable soft-tissue detail is typically limited to a few 100 µm for laboratory systems. Consequently, cellular-sized soft-tissue features like the fibrous cap or foam cells are not observable. Synchrotron-based systems^10,17–19^ have demonstrated somewhat better detail (several tens of micrometers) and contrast but naturally lack the accessibility of the laboratory systems.

X-ray phase contrast^21^ is especially beneficial compared to absorption contrast for observing soft tissues since the phase shifts are three orders of magnitude stronger than absorption in such tissues^14^. Several techniques enable phase-contrast imaging, e.g., interferometry, analyzer-based imaging, grating-based imaging (GBI) and propagation-based imaging (PBI). GBI and PBI can be realized on a laboratory system, with broad-band compact sources. To a first approximation, the generated signal from the phase shift in the object (ϕ) is proportional to the transverse gradient of the phase (∇ϕ) in GBI^22^, and to the transverse Laplacian of the phase (∇^2^ϕ) in PBI^23^. As of today, GBI provides better contrast for low-spatial-resolution imaging, but laboratory PBI with a microfocus source appears superior for high-resolution imaging^24^. Microfocus-source-based PBI has demonstrated cellular-detail bio-imaging by exploiting the high contrast due to air-tissue density differences in carefully prepared samples like excised mouse lungs^25^ and dried mouse brain^26^, and subcellular muscle-fiber contrast in long-exposure imaging of well-defined, uniform and sub-mm-sized zebrafish samples^27^.

In the present paper we show that laboratory propagation-based x-ray phase-contrast tomography provides cellular spatial resolution with excellent contrast in intact autopsy samples of human coronary arteries. Despite the complex multi-material nature of the sample, the method allows imaging of clinically relevant soft-tissue details down to the cellular level, including, e.g., the demarcation of microscopic lipid-rich lesions from its surrounding tissue, observation few foam cells in the vascular wall, and detection of the thin fibrous cap. The tomography is performed on the intact, unstained, formalin-fixed centimeter-sized specimen with a microfocus liquid-metal-jet source for shorter exposure times. We verify the x-ray virtual histology with classical histology and find excellent correspondence. Compared to the classical histology the x-ray tomography has the advantages of being fast, non-destructive, shape preserving and providing 3D data with isotropic resolution in three dimensions.

## Results

### Propagation-based x-ray phase-contrast tomography of coronary artery samples

**Figure 1:**
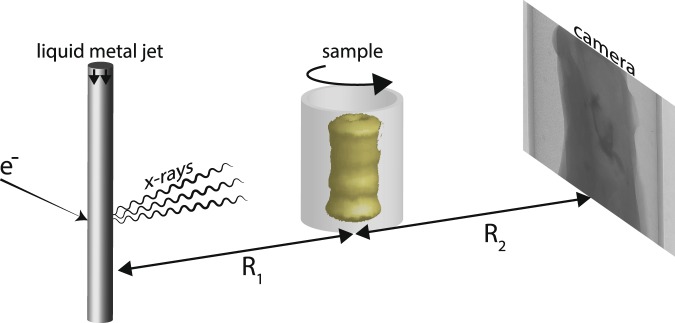
Propagation-based x-ray phase-contrast tomography of human coronary artery samples. The experimental setup consists of an electron-impact liquid-metal-jet microfocus x-ray source, the sample inside a plastic container on a rotating stage, and a high-resolution detector. The geometrical magnification of the object to the detector help achieve higher spatial resolution at the object than at the detector itself. The source-object distance (R_1_) and object-detector distance (R_2_) are marked.

Figure 1 shows the experimental arrangement. It relies on a liquid-metal-jet microfocus x-ray source, a high-resolution scintillator coupled x-ray camera and the sample on a rotating stage. The geometry depends on the source-object distance (*R*_1_) and the object-detector distance (*R*_2_). It magnifies the object to the detector by $M=({R}_{1}+{R}_{2})/{R}_{1}$enabling imaging object resolutions better than the detector resolution. The phase contrast is achieved from an effective propagation distance $\,{z}_{eff}={R}_{1}{R}_{2}/({R}_{1}+{R}_{2})$, due to the divergent cone beam. *M* and *z*_*eff*_ can be set independently by changing *R*_1_ and *R*_2_, and were adjusted to obtain good phase contrast and signal-to-noise ratio^28^ while still keeping the full width of the sample in the field of view. In the present study we optimize the system to allow for detection with cellular resolution, i.e., the 10-µm range, while still maintaining exposure times reasonable. Further details are given in the Methods section.

### Coronary artery sample

**Figure 2:**
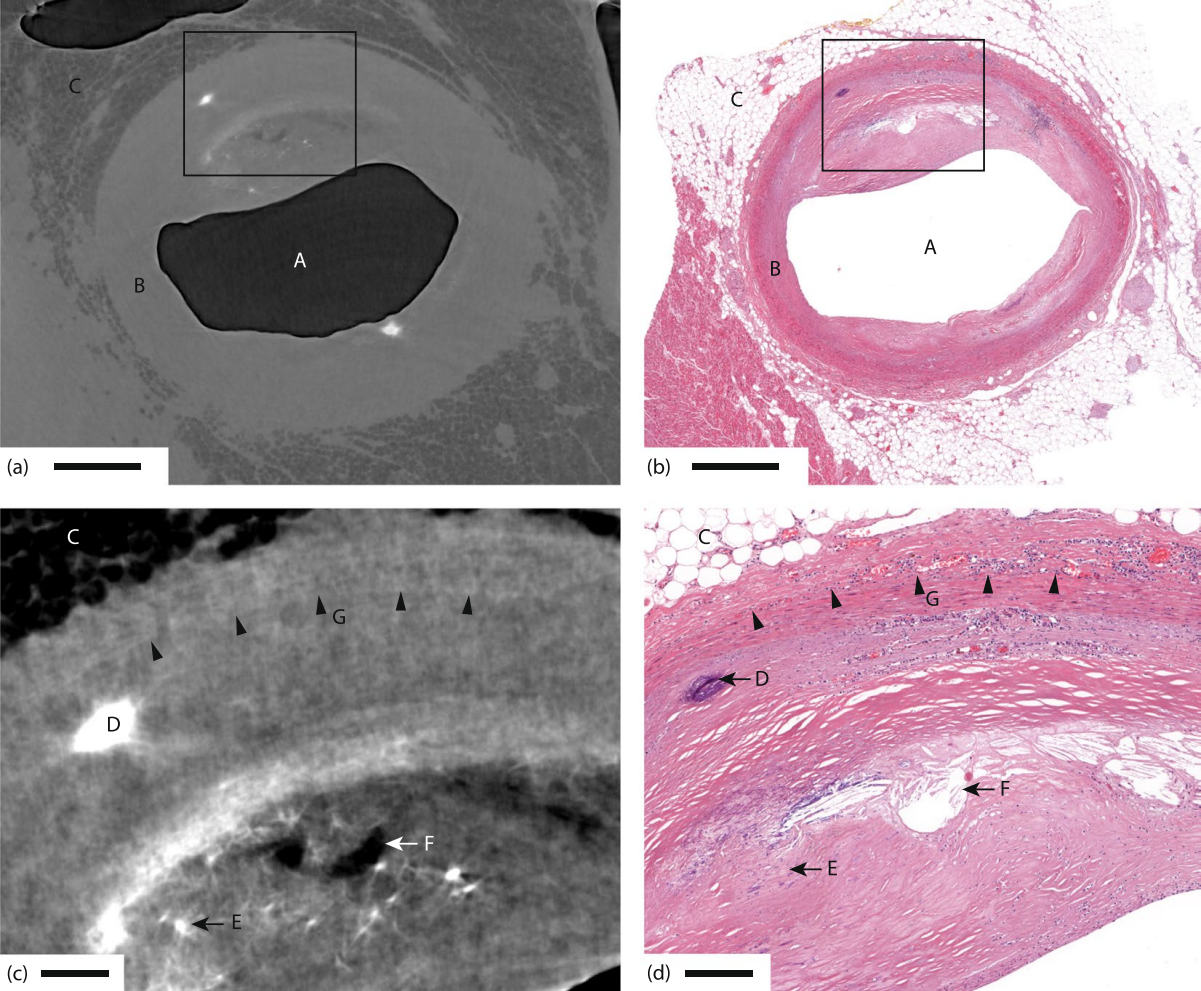
Comparison of propagation-based x-ray phase-contrast tomography and histology for coronary artery sample. (**a**) Section through the x-ray phase tomography volume. (**b**) Histology corresponding to same area as in (**a**). (**c**,**d**) The enlarged areas shown in (**c**,**d**) correspond to the boxes in (**a**,**b**), respectively. A marks the air-filled artery lumen, B the artery wall, C adipose tissue with ∼50 µm adipose cells, D a calcification, E a microcalcification, F cholesterol crystal depositions, and G five arrow-heads indicating the demarcation between tunica media and tunica adventitia. Scale bars are (**a**,**b**) 1 mm, (**c**,**d**) 200 µm.

Figure 2 shows an overview of a typical autopsy sample, illustrating a selection of anatomical features in the propagation-based x-ray phase-contrast tomography and comparing them to their appearance in classical histology. First we note the significant overall similarity between the x-ray phase tomography images and the corresponding histology, e.g., as regards lumen (A) artery wall (B) and adipose tissue (C). In addition we observe calcifications (D and E) and cholesterol crystal depositions (F). The x-ray phase contrast relies on the object density and atomic number. Thus, calcifications with their higher densities and higher atomic numbers will appear brighter, whereas fatty areas appear darker because of the lower density. The calcifications and cholesterol depositions give good contrast towards the surrounding smooth muscle tissue that allow observations down to sub-20 µm features. We also note that individual adipose cells (approx. 50 µm diameter) are resolved, cf. area C in Fig. 2c,d. Distinguishing between smooth muscle tissue, fibrous tissue and tissue with edema is more difficult, because of their similarity in density. In this sample it is possible to identify the border between tunica media and tunica adventitia, despite the low contrast (G).

### Coronary artery sample with severe calcifications

**Figure 3:**
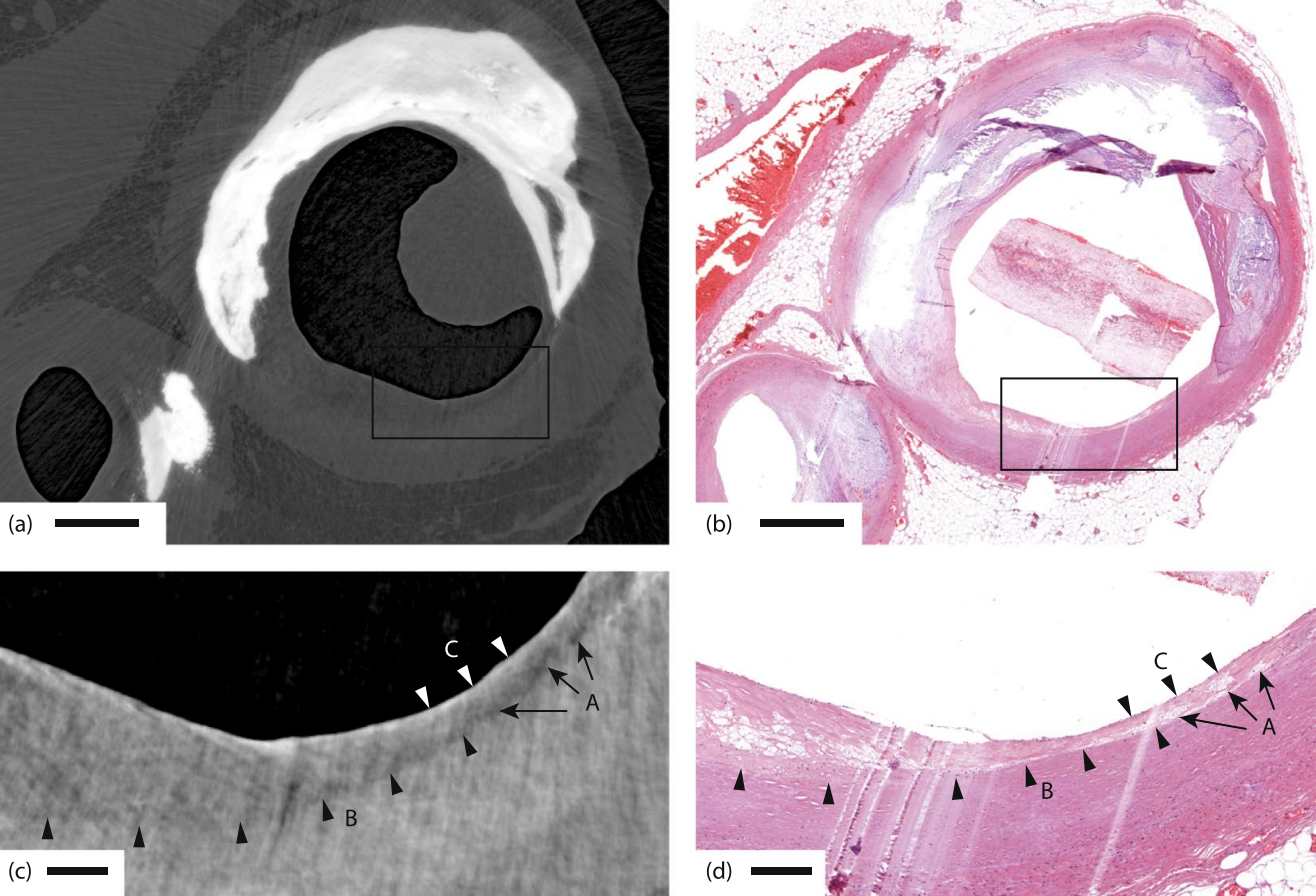
Coronary artery with severe calcifications and lipid lesions. (**a**) X-ray phase tomography. (**b**) Histology of the same sample, resulting in significant distortion due to washing and sectioning. (**c**,**d**) The enlarged areas shown in (**c**,**d**) correspond to the boxes in (**a**,**b**), respectively. A marks three groups of foam cells, B a sharp demarcation from the smooth muscle tissue to a lipid-rich lesion, and C a thin fibrous cap. A, B, and C are clearly observable in both (**c**,**d**). Scale bars are (**a**,**b**) 1 mm, (**c**,**d**) 200 µm.

Figure 3 depicts the comparison between the propagation-based x-ray phase tomography and histology for a sample with severe calcifications. Here the classical histology has difficulties because non-destructive sectioning requires decalcification that leads to washout of calcium deposits. Even small amounts of calcium remnants can cause mechanical sectioning artifacts. The x-ray phase tomography does not require sectioning or removal of calcifications, resulting in a proper representation of the sample’s shape and structure (Fig. 3a), except for some weak streaking artifacts due to the highly absorbing calcifications. In the histology (Fig. 3b), the sample is clearly distorted, both due to the sectioning and due to removal of calcifications. A trained pathologist can still interpret the distorted image, but the x-ray image enables other pathological features to be investigated, such as the accurate measurements of lumen shape and size, also in three dimensions (3D). A 3D-rendering of the blood vessels and calcifications in this sample is available in the Supplementary material.

### Lipid-rich plaques, foam cells, and the thin fibrous cap

**Figure 4:**
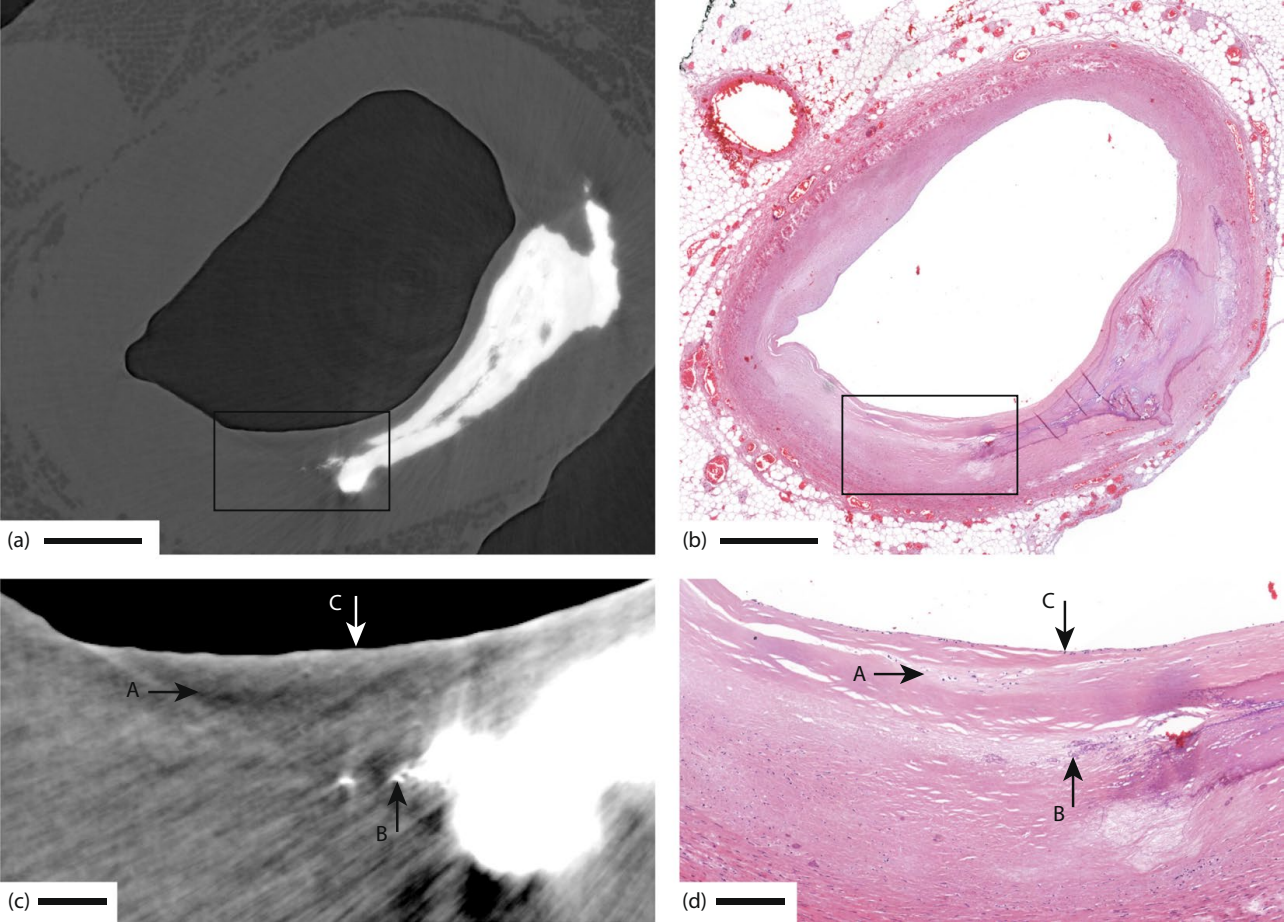
Coronary artery with foam cells. (**a**) X-ray phase tomography image. (**b**) Histology of same area as in (**a**). (**c**,**d**) The enlarged areas shown in (**c**,**d**) correspond to the boxes in (**a**,**b**), respectively. A marks live foam cells, appearing as a dark patch in the x-ray, B the remnants of dead foam cells with incipient calcification (bright dots in x-ray), and C a thin fibrous cap. Scale bars are (**a**,**b**) 1 mm, (**c**,**d**) 200 µm.

Important soft-tissue features such as lipids and foam cells provide much weaker contrast to the surrounding tissue compared to, e.g., calcium-rich plaques, and are therefore difficult to observe in x-ray imaging, especially at high resolution. Despite the weak contrast, the x-ray phase tomography demonstrated here allows such soft-tissue discrimination. In Fig. 3c, B marks a sharp demarcation from the light (smooth muscle) tissue to a darker, i.e., lipid-rich, lesion. In Fig. 3c we also observe three darker spots (A) sized from 20 µm width to several tens of µm length, indicating cellular-sized objects with a lower-density composition. In Fig. 3d these three objects are identified as three groups of foam cells, with a few foam cells in each group. Figure 4c depicts the same type of observations of cellular-sized darker and lighter areas, which are identified as live (A) and dead (B) foam cells in Fig. 4d.

Between the lesion and the lumen in both Figs 3 and 4, there is a thin fibrous cap (C) whose thickness is assumed to be important for assessing the vulnerability. From the x-ray images it is possible to get a rough quantitative measure of its thickness, resulting in less than 25–30 µm in both images. We note that careful data-treatment is necessary to avoid artifacts in the weak-contrast soft-tissue reconstruction, especially when strongly absorbing calcium rich plaques are present in the same sample (cf. Discussion).

## Discussion

We have demonstrated that propagation-based x-ray phase-contrast tomography allows cellular-resolution characterization of intact human coronary artery autopsy samples. This non-destructive method provides high spatial resolution in all three dimensions and gives natural contrast on non-stained samples also for the important lipid-tissue interfaces and allows observation of individual adipose cells, ensembles of few foam cells and possibly single foam cells if they appear in isolation, and the thin fibrous cap. These features are important for the assessment of coronary artery disease and the vulnerability of the plaques. Finally, we note that the method is based on a laboratory microfocus source, making it accessible in the normal histology laboratory.

The key reason for the improved x-ray virtual histology demonstrated here is the combination of high spatial resolution (approx. 10 µm) and high contrast via the phase imaging. Still, the observability of few-ten µm details with low contrast depend also on other factors, especially image noise and reconstruction artifacts. The noise is ultimately photon-limited and can therefore be reduced by increasing exposure times or improving detector efficiency. The reconstruction artifacts are typically more important than the noise and require detailed attention since the coronary artery samples have intrinsic properties making the image reconstruction computationally non-trivial for propagation-based x-ray imaging. For example, to detect lipid-rich plaques also in the vicinity of strongly absorbing calcium-rich plaques, the choice of phase retrieval method is crucial. In the present paper, we used Paganin’s single-material phase retrieval method^29^. Here the processing relies on the object consisting of only one material, albeit at varying density, which obviously does not include calcifications. The phase retrieval was optimized to give best contrast for the lipid-muscle interface, meaning that it slightly over-retrieves (blurs out) the muscle-calcium interface, and slightly under-retrieves (leaves remaining edge enhancement) at the air-muscle interface, since these interfaces have different β/δ-ratio. We chose the established Paganin method for this work in order to avoid erroneous classifications in the virtual x-ray histology since artifacts from the phase retrieval are easily identified as blurring or edge enhancement. However, recently developed phase retrieval algorithms based on interface correction^30–32^ can generalize the phase-retrieval concept to several materials by including rather relaxed assumption about the object. Such multi-material methods could further improve image quality in this type of sample, although they naturally come at the cost of higher computational complexity.

In addition to better algorithms, the proof-of-concept virtual histology arrangement presented here can be improved for higher spatial resolution as well as shorter data acquisition times. Higher resolution requires optimizing the physical experimental arrangement. Operating with a smaller source size increases not only resolution but also contrast for high-spatial-frequency objects, as shown in ref.^27^. An optimal combination of source size and magnification should lead to a few-micron-resolution system. Exploiting such high resolution will require further tailoring of the computational methods for this sample type. As for the speed, we expect the present 7.5 h data acquisition time to be reduced to less than a 1 hour with higher-power liquid-metal-jet sources^25^ and a new detector. We note that the formalin fixation is not necessary for the x-ray imaging, but only if the sample is to be preserved for further studies. Image reconstruction could technically start already during data acquisition, and the final images could thus be ready shortly after the acquisition is finished. Compared to classical histology, which takes several days or weeks, phase contrast tomography can therefore provide much faster results.

In summary: Compared to classical histology on human coronary artery samples, the propagation-based x-ray phase tomography demonstrated here holds several advantages: speed, 3D isotropic high resolution, full 3D dataset for modelling, and less destructive and much simplified sample preparation. It fills the gap between classical CT (3D and fast, but low resolution) and classical histology (2D and slow, but still with higher resolution in the sections). The rapid and non-destructive x-ray tissue characterization opens up for virtual histology on large sets of coronary artery samples in full 3D. The high and isotropic 3D resolution can be used for identifying interesting areas/volumes for subsequent spatially targeted detailed analysis with classical histology. We note that *in vivo* thin-cap imaging with the detail demonstrated here would be a valuable clinical tool. However, this is a very different imaging task and we therefore refrain from speculating on the potential of the method. As for tissue analysis in general, the method should be applicable also outside that of coronary artery samples. The strong natural contrast provides discrimination between, e.g., calcifications, fat, and different tissue types with high spatial resolution, making the method potentially applicable for a vast range of tissue types and histo-pathological examinations. For example, dystrophic calcifications are common in human pathology in many conditions associated with inflammation, infection and malignant tumors. Concentric depositions of calcium salts (psammoma bodies) in various tumors have special diagnostic value and are just on the border of detection with current CT. Fatty degeneration of various organs due to metabolic lesions (liver steatosis), chronic inflammation (Crohn’s disease of the gut) or aging (bone marrow) are other important conditions where high resolution quantitative 3D imaging may contribute to the understanding of the underlying biological processes.

## Methods

### Human autopsy samples

Human coronary arteries together with some of the surrounding tissue, were excised post mortem from patients likely to have extensive atherosclerosis. The samples were cut into 2–3 cm long pieces and fixed in formalin. All samples were anonymized and handled in accordance with the ethical permit (Regionala Etikprövningsnämnden in Stockholm 2015/684-31/2). 5 samples from 2 persons were used.

### Propagation-based x-ray phase-contrast tomography arrangement

The arrangement for propagation-based x-ray phase-contrast tomography^14,21,23^ is depicted in Fig. 1. The setup consists of a microfocus x-ray source, a rotation stage for the autopsy sample and a high-resolution x-ray camera. As x-ray source, we used a MetalJet D2 (Excillum AB, Sweden) operated at 60 kV and 68 W, focused to a 8×32 µm spot on the galinstan jet, resulting in a 8×8 µm x-ray spot. The spectrum is dominated by the 9.2 keV Ga emission line. The rotation stage was a Newport URS50BCC (Newport, California) and the detector a Photonic Science VHR (Photonic Science, UK) CCD detector with 4008×2671 pixels with a pitch of 9 µm, fiber-optically coupled (1:1) to a 15 µm gadolinium oxysulfide scintillator. The detector point-spread function had a full-width-half-maximum (FWHM) of 27 µm.

### Data acquisition

The samples were immobilized in a 10-mm diameter plastic tube, which was sealed in both ends to maintain a humid environment around the sample. The source-object-distance was 44.5 cm, and the source-detector-distance 100 cm. Thus, the magnification was *M* = *2*.*25*, resulting in a 4.0 µm pixel size in the object plane. The detector-limited resolution in the object plane is 12 µm. For each tomographic dataset, 1800 projections were recorded over 180° with an exposure time of 15 s per projection. The total exposure time was 7.5 hours and the dose is approx. 2.6 Gy average in the soft tissue.

### X-ray image reconstruction

The projection images were flat-field corrected, and phase retrieved using Paganin’s method^29^, with β/δ = 0.0045 and λ = 0.124 nm. The phase-retrieved images were tomographically reconstructed using Octopus Reconstruction (Inside Matters NV, Belgium). Grey scales have been adjusted for each image.

### Histology

The samples were fixed in formalin prior to decalcification, dehydration, paraffin embedding, microtome sectioning and hematoxylin eosin staining using routine clinical histopathology procedures. The stained and mounted specimens were scanned using automated slide scanner (Panoramic SCAN, 3DHistech, Budapest, Hungary).

### Image matching and analysis

The three-dimensional x-ray image stacks were analyzed by an experienced medical doctor (J.P.) and a physicist (W.V.), to find the best match with the histology images, using anatomical landmarks. All four authors (two MD and two physicists) evaluated the matched images to find, classify and analyze structures.

### Data availability

Full imaging datasets are available upon reasonable request to the corresponding author.

## Electronic supplementary material

Supplementary information

Video 1

## References

[1] WHO Global Health Observatory (GHO) data report. Health in 2015: from MDGs to SDGs. Geneva: World Health Organisation; 2015 (http://apps.who.int/iris/bitstream/10665/200009/1/9789241565110_eng.pdf?ua=1, accessed 18^th^ of June, 2017)

[2] Hansson GK, Libby P (2006). The immune response in atherosclerosis: a double-edged sword. Nat. Rev. Immunol..

[3] Falk E (1983). Plaque rupture with severe pre-existing stenosis precipitating coronary thrombosis. Characteristics of coronary atherosclerotic plaques underlying fatal occlusive thrombi. Br. Heart. J..

[4] Virmani R, Kolodgie FD, Burke AP, Farb A, Schwartz SM (2000). Lessons from sudden coronary death: a comprehensive morphological classification scheme for atherosclerotic lesions. Arterioscler. Thromb. Vasc. Biol..

[5] Narula J (2013). Histopathologic characteristics of atherosclerotic coronary disease and implications of the findings for the invasive and noninvasive detection of vulnerable plaques. J Am Coll Cardiol..

[6] Bom MJ (2017). Early Detection and Treatment of the Vulnerable Coronary Plaque: Can We Prevent Acute Coronary Syndromes?. Circ. Cardiovasc. Im..

[7] Kramer MC (2010). Relationship of thrombus healing to underlying plaque morphology in sudden coronary death. J. Am. Coll. Cardiol..

[8] Hillman H (2000). Limitations of clinical and biological histology. Med. Hypotheses.

[9] Langheinrich AC (2014). Atherosclerotic Lesions at Micro CT: Feasibility for Analysis of Coronary Artery Wall in Autopsy Specimens. Radiology.

[10] Holme MN (2014). Complementary X-ray tomography techniques for histology-validated 3D imaging of soft and hard tissues using plaque-containing blood vessels a examples. Nature Prot..

[11] Kelly-Arnold A (2013). Revised microcalcification hypothesis for fibrous cap rupture in human coronary arteries. Proc. Nat. Acad. Sci..

[12] Matlung HL (2009). Calcification Locates to Transglutaminases in Advanced Human Atherosclerotic Lesions. Am. J. Pathol..

[13] Wintermark M (2008). High-resolution CT imaging of Carotid Artery Atherosclerotic Plaques. AJNR Am. J. Neuroradiol..

[14] Bravin A, Coan P, Suortti P (2013). X-ray phase-contrast imaging: from pre-clinical applications towards clinics. Phys. Med. Biol..

[15] Hetterich H (2015). X-ray Phase-Contrast Computed Tomography of Human Coronary Arteries. Invest. Radiol..

[16] Hetterich H (2016). HA classification of coronary and carotid atherosclerotic plaques by grating-based phase-contrast computer tomography. Eur. Radiol..

[17] Buscema M (2016). Histology-validated x-ray tomography for imaging human coronary arteries. Proc. SPIE.

[18] Hetterich H (2013). Grating-based X-ray phase-contrast tomography of atherosclerotic plaque at high photon energies. Z. Med. Phys..

[19] Saam T (2013). Translation of Atherosclerotic Plaque Phase-Contrast CT Imaging from Synchrotron Radiation to a Conventional Lab-Based X-Ray Source. PLoS ONE.

[20] Hetterich H (2014). Phase-Contrast CT: Qualitative and Quantitative Evaluation of Atherosclerotic Carotid Artery Plaque. Radiology.

[21] Fitzgerald R (2000). Phase-Sensitive X-Ray Imaging. Phys. Today.

[22] Pfeiffer F, Weitkamp T, Bunk O, David C (2006). Phase retrieval and differential phase-contrast imaging with low-brilliance X-ray sources. Nat. Phys..

[23] Wilkins SW, Gureyev TE, Gao D, Pogany A, Stevenson AW (1996). Phase-contrast imaging using polychromatic hard X-rays. Nature.

[24] Zhou T (2013). Comparison of two x-ray phase-contrast imaging methods with a microfocus source. Opt. Express.

[25] Larsson DH, Vågberg W, Yaroshenko A, Yildirim AÖ, Hertz HM (2016). High-resolution short-exposure small-animal laboratory x-ray phase-contrast tomography. Sci. Rep..

[26] Töpperwien M (2017). Three-dimensional mouse brain cytoarchitecture revealed by laboratory-based x-ray phase-contrast tomography. Sci. Rep..

[27] Vågberg W, Larsson DH, Li M, Arner A, Hertz HM (2015). X-ray phase-contrast tomography for high-spatial-resolution zebrafish muscle imaging. Sci. Rep..

[28] Mayo SC (2002). Quantitative X-ray projection microscopy: phase-contrast and multi-spectral imaging. J. Microsc..

[29] Paganin D, Mayo SC, Gureyev TE, Miller PR, Wilkins SW (2002). Simultaneous phase and amplitude extraction from a single defocused image of a homogeneous object. J. Microsc..

[30] Ullherr M, Zabler S (2015). Correcting multi material artifacts from single-material phase retrieved holo-tomograms with a simple 3D Fourier method. Opt. Express.

[31] Beltran MA, Paganin DM, Uesugi K, Kitchen MJ (2010). 2D and 3D X-ray phase retrieval of multi-material objects using a single defocus distance. Opt. Express.

[32] Häggmark I, Vågberg W, Hertz HM, Burvall A (2017). Comparison of quantitative multi-material phase retrieval algorithms in propagation-based phase-contrast X-ray tomography. Opt. Express.

