# Supplementary material for: Cellular-resolution 3D virtual histology of human coronary arteries using x-ray phase tomography
Source: Sci Rep. 2018 Jul 20;8:11014. doi: 10.1038/s41598-018-29344-3 (PMC6054690; doi:10.1038/s41598-018-29344-3)
Supplement: Supplementary file 1 — Supplementary information [file 41598_2018_29344_MOESM1_ESM.docx]

SUPPLEMENTARY MATERIAL

Cellular-resolution 3D virtual histology of human coronary arteries using x-ray phase tomography

William Vågberg^1^*, Jonas Persson^2^, Laszlo Szekely^3,4^, and Hans M. Hertz^1^

^1^ Department of Applied Physics, KTH Royal Institute of Technology/Albanova, Stockholm, Sweden

^2^ Karolinska Institutet, Division of Cardiovascular Medicine, Department of Clinical Sciences, Danderyd University Hospital, Stockholm, Sweden

^3^ Laboratory of Clinical Pathology and Cytology, Karolinska University Hospital, Stockholm, Sweden

^4^ Department of Medicine, Solna, Karolinska Institutet, Stockholm, Sweden.

*Correspondence: [william.vagberg@biox.kth.se](mailto:william.vagberg@biox.kth.se)

**Video 1. 3D reconstruction of coronary artery bifurcation with calcifications.** This video corresponds to the dataset of Fig. 3. The original voxels are binned 3×3×3 to produce 12 µm voxels.

September 26, 2017
